# Cytokines secreted from bone marrow derived mesenchymal stem cells promote apoptosis and change cell cycle distribution of K562 cell line as clinical agent in cell transplantation

**DOI:** 10.1371/journal.pone.0215678

**Published:** 2019-04-22

**Authors:** Ezzatollah Fathi, Raheleh Farahzadi, Behnaz Valipour, Zohreh Sanaat

**Affiliations:** 1 Department of Clinical Sciences, Faculty of Veterinary Medicine, University of Tabriz, Tabriz, Iran; 2 Hematology and Oncology Research Center, Tabriz University of Medical Sciences, Tabriz, Iran; 3 Stem Cell Research Center, Tabriz University of Medical Sciences, Tabriz, Iran; Università degli Studi della Campania, ITALY

## Abstract

Mesenchymal stem cells (MSCs) are of special interest due their potential clinical use in cell-based therapy. Therapies engaging MSCs are showing increasing promise in the cancer treatment and anticancer drug screening applications. A multitude of growth factors and cytokines secreted from these cells are known to give such multifunctional properties, but details of their role are yet to be absolutely demonstrated. In this study, we have evaluated the influence of BMSCs on K562 cell line as chronic myeloid leukemia (CML) cells, with the use of a cytokine antibody array recognizing 34 cytokines. For this purpose, BMSCs were isolated and co-cultured with K562 cells; thereafter, cultured K562 alone and co-cultured K562 with BMSCs (10:1) were collected at day 7 and subjected to cell cycle distribution assay as well as annexin/PI analysis and Ki/caspase-3 assay for apoptosis assessment. In the following, the gene and protein expression levels of BAX and BCL-2 as pro- and anti-apoptotic agents were investigated. Furthermore, after 7 days’ treatment, culture medium was collected from both control and experimental groups for cytokine antibody array. It was found that BMSCs resulted in a robust increase in the number of cells at G_0_/G_1_ phase and arrest the G_0_/G_1_ phase as well as significantly inducing late apoptosis in K562 cells. The significant presence of TIMP-1 (tissue inhibitor of metalloproteinases-1), and moderate elevated signals for CINC-1 (cytokine-induced neutrophil chemoattractant-1) were obvious in the co-cultured conditioned media, but no significant increase was found in 32 other cytokines. It is concluded that co-culture of BMSCs with K562 cells could secrete a substantial amount of TIMP-1 and CINC-1. These cytokines could be involved in the inhibition of the K562 cell proliferation via BAX and caspase-3 cascade pathways.

## Introduction

Mesenchymal stem cells (MSCs), which are present in adult organs and tissues such as heart, liver, kidney, adipose tissue, bone marrow, placenta, amniotic fluid, amnion, etc., are undifferentiated multipotential cells that have the capacity to differentiate into a broad range of different cell types, including osteocytes, adipocytes, chondrocytes, neuron-like cells and other connective tissues [1–4]. Also, due to the self-renewal, plasticity and relatively non-immunogenic properties, MSCs are potentially responsible for transplantation, regeneration and treatment of some diseases such as ischemia, stroke, multiple sclerosis, cardiac events, cartilage and bone pathologies, auto-immune disorders, cancer, blood malignancy and genetic diseases [5, 6]. From the mentioned diseases, hematological abnormality and blood malignancy have gained more attention for cell transplantation with MSCs. Numerous studies have been conducted with bone marrow derived-MSCs (BMSCs) and there are no reports of tumor formation after transplantation with BMSCs which is the same in other animal and human sources. In addition, it was reported that BMSCs could favor tumor growth either by enhancing tumor cells invasive abilities or by protecting them from immune cell recognition [7]. In the other words, there are concerns about these cells and the risks linked to cell treatment still remain unclear, particularly in the context of patients affected by pre-existing cancer [8]. It was reported that interactions between cancer cells and MSCs are of fundamental importance in stimulating both the development and invasiveness of tumors [9]. For example, tumor cells may lead to modifications of surveying and molecular composition of MSCs as stroma cells during tumor development and this, can affect the cancer cells properties [10]. Therefore, the bidirectional interplay between tumor cells and MSCs, plays an important role in tumor progression and invasion and creates a complex microenvironment called tumor niche. Fibroblasts as normal stroma, are predominant cells that secrete an extracellular matrix (ECM) providing a natural barrier against tumor progression [11]. In these processes, MSCs can be basic. It has been indicated that MSCs can originate from tumor resident stroma progenitor cells [12]. Interestingly, MSCs have the potency to migrate into damaged tissues, driven by chemotactic gradients of cytokines released from same damaged tissues [13]. However, others have found the opposite [14]. Various studies have been conducted to examine the effect of MSCs on proliferation, growth and the percentage of apoptosis of cancer cell line [15]. For example, in one study, Zhang (2009) reported that co-culture of MSCs with CML extracted from bone marrow of newly diagnosed patients could secrete a substantial amount of IFN-α, thus inhibiting the proliferation of CML cells [16]. In another study, Fonseka et al. (2012) indicated that umbilical cord blood-derived mesenchymal stem cells could inhibit the proliferation of K562 cell line due to arrest in the G0/G1 phase as well as increase in the IL-6 and IL-8 secretion and LAP (latency-associated peptide; TGFb1) [17]. On the other hand, it was shown that BMSCs could mediate immunosuppression via secreting soluble cytokines [16]. But there are rare reports of the effect of the kind and amount of secreted growth factors and cytokines from BMSCs and the underlying mechanisms. All studies up to now, have shown the effects of MSCs on cancer cells. On the contrary, in one study by Paino et al. (2017), the effects of cancer cells on adipose tissue-derived MSCs differentiation was investigated. It was shown that in the presence of cancer cells, MSCs do not differentiate *in vitro* or facilitate the tumor angiogenesis *in vivo*. These results opening interesting new scenarios in the relationship between cancer and stem cells. These findings may also lead to greater caution, when managing autologous fat grafts in cancer patients [13]. With these explanations, the aim of this study was to explore the influence of BMSCs on proliferation and apoptosis of K562 cell line as chronic myeloid leukemia (CML) cells via investigation of secreted cytokines. For this purpose, cultured K562 alone and co-cultured K562 and MSCs (10:1) were collected at day 7 and subjected to cell cycle and annexin/PI analysis. Also, at the end of the 7th day, supernatant of the two groups of cells was collected for cytokine antibody array.

## Materials and methods

### Reagents

**Fig 1:**
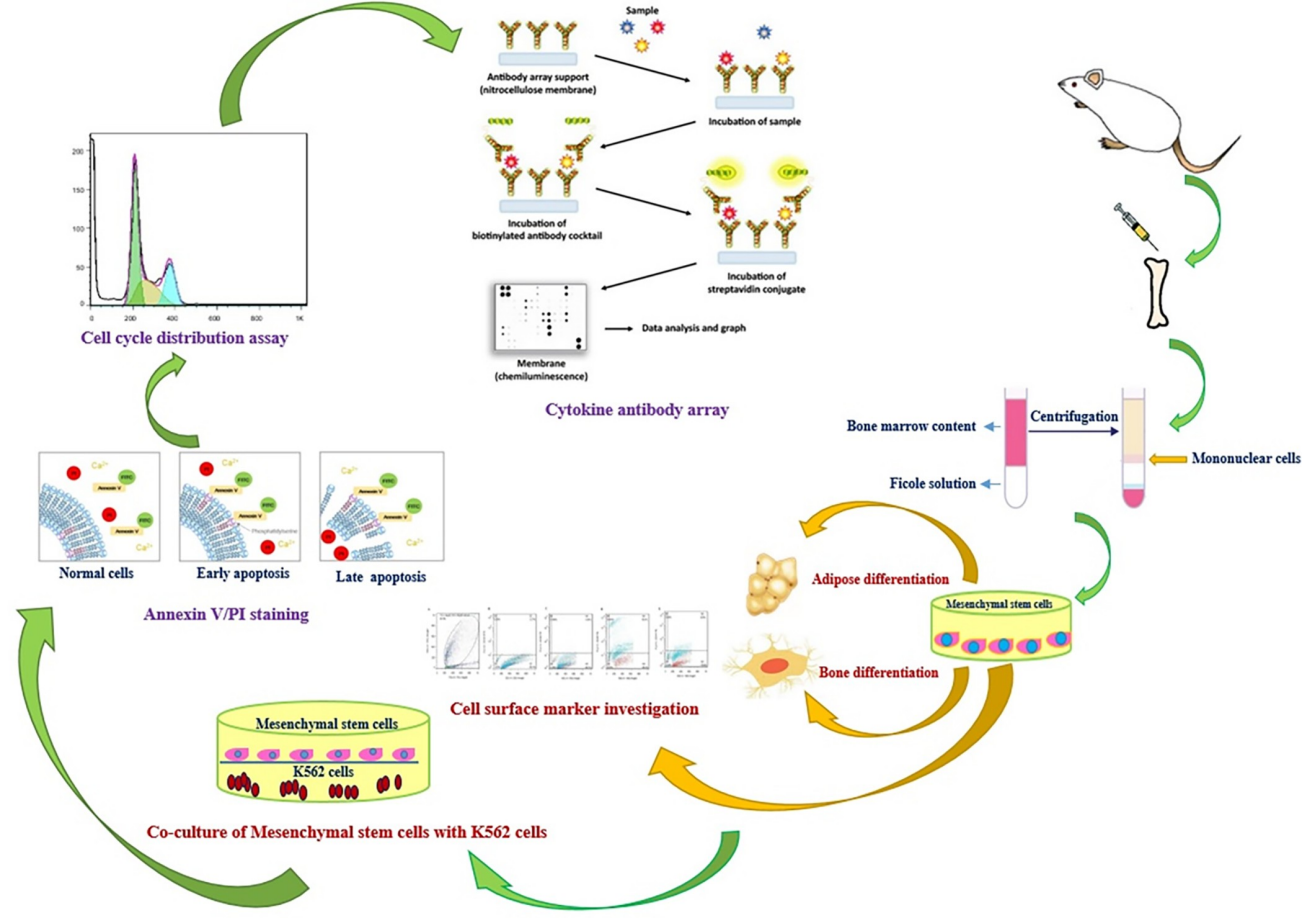
An overview of the experimental procedures that have been done in this paper.

All chemicals and cell culture plates, if not otherwise specified, were purchased from Sigma-Aldrich (Invitrogen, Carlsbad, Calif., USA) and SPL Life Sciences Co., Ltd. (Gyeonggi-do, Korea), respectively. All experimental procedures were repeated three times. A comprehensive overview of methods that have been used in this paper was described as Fig 1. All the used primers are listed in Table 1.

**Table 1:** Primer sequences used for RT and Real time-PCR.

No.	Gene	Primer pair sequence (5'-3')	Product length (bp)
NM_013059.1	ALP	CCTTGAAAAATGCCCTGAAA	191
		CTTGGAGAGAGCCACAAAGG	
NM_001278484.2	OCN	GTCCCACACAGCAACTGC	219
		CCAAAGGCTGAAGCTGCCG	
NM_013196.1	PPAR-alpha	CCCTGCCTTCCCTGTGAACTGAC	387
		GGGACTCATCTGTACTGGTGGGGAC	
NM_013124.3	PPAR-gamma	GGTGAAACTCTGGGAGATCC	400
		TGAGGGAGTTTGAAGACTCTTC	
NM_017008.4	GAPDH	ATGACTCTA CCCACGGCAAG	88
		CTGGAGATGGTGATGGGTT	
NM_138761.4	BAX	TGCCAGCAAACTGGTGCTCA	194
		GCACTCCCGCCACAAAGATG	
NM_000633.2	BCL2	TCTGTGGATGACTGAGTACCTGAAC	129
		AGAGACAGCCAGGAGAAATCAAA	

### Isolation of MSCs from bone marrow

In this research, ethical consent was specifically approved by an animal ethics committee at Tabriz University of Medical Sciences, Tabriz, Iran (Ethic Code No: IR.TBZMED.REC.1396.849) in accordance with the guidelines of the World Medical Associations (VMA) Deceleration of Helsinki-Ethical Principles for Medical Research regarding experiments performed on animals under the sections of Scientific Requirements and Research Protocols (paragraph 21) and Research Ethics Committees (paragraph 23). About 3 (4- to 9-week-old) male Rattus rats were purchased and euthanized using ketamine (87 mg/kg)/xylazine (13 mg/kg). Bone marrow from the tibia and femur was obtained by flushing with 26-gauge syringe needle containing phosphate-buffered saline (PBS) supplemented with 5% fetal bovine serum (FBS) (washing buffer) in a sterilized culture dish. Bone marrow contents were washed with washing buffer and centrifuged at 360×g (1500 rpm) for 5 min. In the following, 10 mL of cell suspension was layered over 10 ml of Ficoll-Paque (Baharafshan, Tehran, Iran) in a 15-mL falcon tube and centrifuged at 850×g (2298 rpm) for 25 min at 4°C. Thereafter, middle phase containing mononuclear cell layer was transferred to a new 15-mL falcon tube and was washed twice with washing buffer. The cell pellet was re-suspended in Dulbecco’s modified Eagle’s medium (DMEM, Gibco, UK) containing 10% (v/v) FBS and 1% (v/v) penicillin/streptomycin solution (complete culture medium). Cell cultures were maintained in a 37°C incubator with 5% CO_2_ and passaged with 0.25% trypsin (Gibco, UK) and 1 mM ethylenediaminetetraacetic acid (EDTA; Invitrogen, UK) when required. The medium was replaced twice weekly during the cells cultivation. Cells of passages 3–6 were used throughout the present study [18].

### Multi-lineage differentiation of MSCs derived bone marrow

The BMSCs were grown to confluence and a multi-lineage differentiation of cells was induced as previously described by Farahzadi et al., (2017) [19]. In brief, the cells were cultured in one of the following two induction media as follows: (1) adipogenesis medium: 10 μg/ml insulin, 1μM dexamethasone, 0.5 mM 1-methyl-3 isobutylxanthine and 200 μM indomethacin; (2) osteogenesis medium: 10 mM b-glycerophosphate, 10 nM dexamethasone and 0.05 mM L-ascorbic acid-2-phosphate. At the end of the 21st day, the cells were fixed with 4% (v/v) paraformaldehyde for appropriate differentiation-specific staining: Sudan III (1% in 96% ethanol) for adipogenesis and Alizarin red (2% in distilled water) for osteogenesis. Also, the expression of PPAR-α and PPAR-γ as adipocyte-specific genes as well as the expression of ALP and OCN as bone-specific genes were detected by RT-PCR as previously described by Mobarak et al. (2017) [20].

### Characterization of MSCs derived bone marrow

Characterization of mesenchymal stem cells (MSCs) was done by flow cytometry as previously described by Farahzadi et al. (2017) [19]. In brief, approximately 10×10^5^ bone marrow derived-MSCs from the passage 4 cultures were collected and incubated with an appropriate amount of fluorescein isothiocyanate (FITC)-conjugated antibody CD34 and phycoerythrin (PE)–conjugated CD44, CD56 and CD90 (BD Phar-mingen, San Diego, CA, USA) (1μg/10^6^cells) in PBS supplemented with 3–5% FBS (washing buffer) for 30 min on ice. After washing of the cells, fluorescence activated cell sorter (FACS) instrument (Becton Dickinson Franklin Lakes, USA) was used to quantify the fluorescence intensity of BMSCs and the data were analyzed with a FlowJo software (version 6.2) [3, 21].

### Population doubling time (PDT) assessment

To access the exponentially growing cells rate of the MSCs obtained from the bone marrow, PDT (the time required by the cells to double their population) was calculated. For this purpose, BMSCs from passage 4 were seeded into the 12-well plates (20×10^4^ cells/well). At the end of 24, 48, 72, 96, and 120 hours, the cells were collected and counted and PDT was calculated using the following equation: PDT = CT/PDN, where CT is the culture time and PDN the population doubling number. Also, PDN = log (N1/N0) ×3.31. In this equation, N1 is the cell number at the end of cultivation period, N0 is the cell number at culture initiation.

### Cell culture of myelogenous leukemia cell line (K562)

K562 (ATCC CCL-243) as chronic myelogenous leukemia cell line was purchased from Pasteur Institute of Iran. Cells were cultured in suspension in Roswell Park Memorial Institute 1640 (RPMI 1640, Gibco, UK) supplemented with 10% (v/v) FBS and 1% (v/v) penicillin/streptomycin solution [22].

### Co-culture of BMSCs and CML-cell line (K562) in a trans-well system

Cryopreserved K562 was routinely recovered and the number of live cells was counted using Trypan blue staining. The cell density was adjusted to 150×10^3^/cm^2^ using RPMI 1640 containing 10% FBS and 1% (v/v) penicillin/streptomycin solution. BMSCs of passage 3 were detached using trypsin-EDTA, collected and plated into three 6-well plates at 10×10^4^ cells/well in 1 ml DMEM complete culture medium solution. After 24 h, 10×10^5^ cells/well in 2 mL RPMI 1640 complete culture medium solution was added respectively into two BMSCs groups; control group (culture of BMSCs alone) and experimental group (co-cultured K562 and BMSCs). At day 7, cultured K562 alone and co-cultured K562 and MSCs (10:1) were collected and subjected to cell cycle and annexin/PI analysis. Also, at the end of the 7th day, supernatants of the two groups of cells were collected for cytokine antibody array.

### Cytokine secretion profiling by cytokine antibody array

Rat Cytokine Antibody Array–Membrane (ab133992, abcam, USA) consisting of a total of 34 different cytokine antibodies spotted in duplicate onto two membranes was used. For this purpose, supernatants from the experimental group and control group were collected at culture day 7. Array membranes, each in separate wells of provided 8-well plate, were incubated for 30 min in 2 ml of blocking buffer, and further incubated for 2 hours in a shaker at room temperature with 2 ml of fresh culture medium. After the membranes were thoroughly washed with wash buffer I and II, 2 μl of biotin-conjugated antibodies at 1:1000-fold dilution was added to each membrane, and the mixture was incubated on a shaker at 4 ^o^C overnight. Following the wash, the membranes were incubated with a 1:1000 dilution of HRP-conjugated streptavidin for 2 hours at room temperature. Proteins were detected by detection buffer C and D provided in kit and signals were captured by CCD camera. The exposure time was 5 minutes and it was completed within 20 minutes as chemiluminescence signals will fade over time. Arrays images were processed with Image J software.

### Flow cytometric analysis for cell-cycle staining and distribution

This technique was previously described by Cheraghi et al., 2016 [23]. Briefly, K562 cells (20×10^4^ cells/well) from the experimental and control groups were collected, washed twice in cold PBS, fixed by cold ethanol (70% w/w) and then incubated in PBS supplemented with 0.1% Triton X-100, 0.1% sodium citrate and propidium iodide (PI) solution (50 μg/ml; Sigma) at 4°C for 30 min. Finally, the percentage of cells in each phase of the cell cycle, including SubG_1_, G_1_, S and G_2_/M was analyzed by flow cytometry method using a BD FACSCalibur system.

### Flow cytometric detection of apoptosis and necrosis by Annexin V/PI assay

As mentioned above, 20×10^4^ K562 cells/well from the control and experimental groups were trypsinized, blocked by FBS, washed twice with PBS, collected, resuspended in the binding buffer (Ref No: 00-0055-56, ebioscience) and kept for 20 min in the dark at 4 ^o^C. In the following, cells were incubated with 100 μl binding buffer containing 5 μl of FITC-conjugated Annexin V (Ref No: 11-8005-74, ebioscience) for 15 min at 25 ^o^C. Afterward, cells were washed with binding buffer and exposed to PI solution in 100 μl binding buffer. Flow cytometry was performed by FACSCalibur (BD Bioscience), and the data were analyzed with FlowJo software ver. X.0.7 [24].

### Ki-67 proliferation and caspase-3 apoptosis assay

To detect bone marrow derived-MSCs effect on K562 cell line proliferation and caspase apoptosis assessment, K562 cell line was co-cultured with bone marrow derived-MSCs for 7 days. For Ki proliferation assay, following the co-culture period, cells were trypsinized, washed with PBS and incubated with 0.2% Triton X-100 for 15 min and stained with 5 μl of Ki-67 antibody solution (Cat No: 12-5699-42) for 30 min and subsequently analyzed by flow cytometry. In addition, for caspase-3 assay, K562 cells were harvested, and washed twice by washing buffer (PBS supplemented by 5% FBS). These cells were immediately fixed using FCM fixation buffer (sc-3622, Santa Cruz, CA) for 30 min, washed by washing buffer, and permeabilized by FCM permeabilization buffer (sc-3623, Santa Cruz, CA) for 5 min at RT. Washed cells were immediately stained using PE conjugated mouse anti-caspase (BD Bioscience, Germany) for 30 min and analyzed by flow cytometry.

### RNA extraction, cDNA synthesis and quantitative Real time-PCR

At the end of co-culture period, 10×10^5^ K562 cells/well from the control group as well as the experimental group were collected and total RNA was extracted from these cells using Trizol reagent (Invitrogen, UK). Extracted cellular RNA was dissolved in diethyl phosphorocyanidate-treated water and 2 μg RNA was used for the first strand cDNA synthesis in a total volume of 20 μL according to the manufacturer’s guidelines. The mRNA expressions of target genes included BAX, BCL2 and β-actin. All PCR reactions were performed using the Corbett Rotor-Gene 6000 HRM (Corbett Research, Australia) in a total volume of 20 μL containing Power SYBR Green master mix (2x) (TaKaRa Ex Taq HS, Japan), Primer fwd (0.5 μM), Primer rev (0.5 μM), cDNA (30 ng/μl) and H_2_O. The thermal cycling conditions were beginning of the denaturation step for 5 min at 95°C, followed by 40 cycles, each denaturation at 95°C for 10 s, annealing at 60°C (BAX and BCL2) or 59°C (β-actin) for 15 s and extension at 72°C for 20 s. Fluorescence data was analyzed by using Rotor-Gene 6000 Software version to obtain CT values. The CT values were calculated in relation to β-actin CT values by the 2^-ΔΔCT^ method, in which ΔCt was the difference between the Ct value of genes and the Ct value of β-actin [25]. Primers were designed using Oligo 7 v.7.52 software (Molecular Biology Insights, Inc, USA).

### Western blot analysis for BAX and BCL2 protein expression

As mentioned above, at the end of co-culture period, K562 cells were collected, washed twice with cold PBS and lysed using RIPA-buffer for 30 min at 4°C. Homogenates were centrifuged at 13000×g for 15 min at 4°C and protein concentrations were calculated with the BCA protein Assay (Pierce, Rockford, IL, USA). Then, 50 μg of each protein sample was loaded on 12% SDS-PAGE and transferred to poly vinylidene difluoride (PVDF) membrane. The skim milk (5%) in TBS-T (20 mM Tris, 137 mM NaCl and 0.1% Tween 20) was used for blocking of the membranes for 60 min at 25°C. In the following, the membranes were incubated overnight at 4°C with primary polyclonal antibodies against β-actin (1:1000), BAX and Bcl-2 (1:500, Santa Cruz Biotechnology, CA), washed twice with TBS-T and were incubated with goat anti-mouse secondary antibody (1:5000 Santa Cruz) diluted in TBS-T for 60 min at 25°C. Next, the membranes were washed and protein bands were detected using enhanced chemiluminescence detection Kit (Roche, UK) with X-ray film. The intensity of protein bands was measured by ImageJ 1.6 software and signal intensity of each band was normalized to its corresponding β-actin control [26].

### Statistical analysis

The results were analyzed using the software program Graph Pad Prism version 6.01. We used one-way and two-way ANOVA followed by Dunnett’s post hoc test to determine the significant difference among groups. Also, statistical significance was determined at p<0.05. All experimental procedures were repeated for three times.

## Results

### In vitro multi-lineage differentiation and PDT assessment of MSCs derived bone marrow

**Fig 2:**
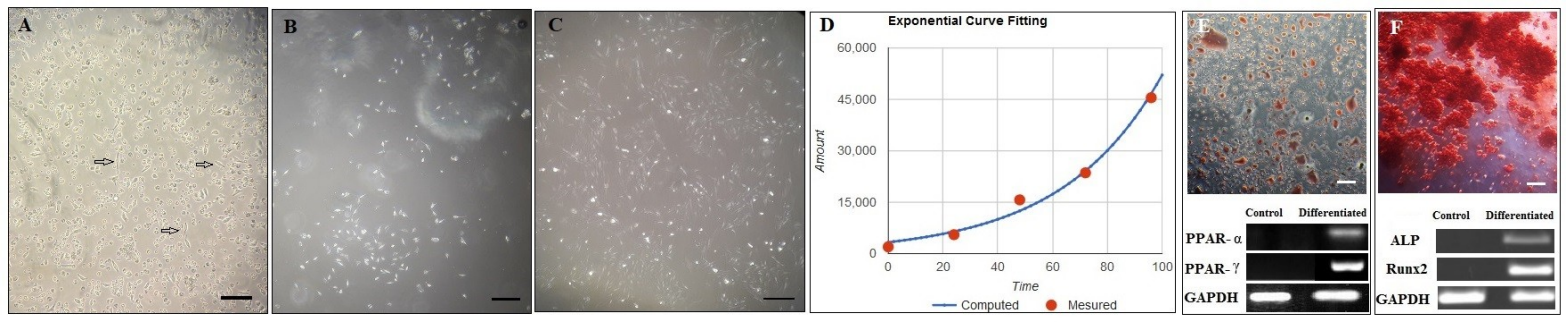
**Morphological features of bone marrow derived-MSCs;** (A) Spindle-shaped morphology of bone marrow cells that appear at day 1, (B) The number and size of colony appears to gradually increase on days 3–7, (C) More confluent bone marrow derived-MSCs at 21 days of culturing cells. (D) **population doubling time (PDT) of bone marrow derived-MSCs. Two-lineage differentiation of bone marrow derived-MSCs;** (E) Generation of lipid vacuoles after adipogenesis and staining by Sudan III; expression of PPAR-α and PPAR-γ as fat-specific genes, (F) Osteogenic differentiation and Alizarin red staining of mineralized cell aggregates; detection of the two bone specific genes (ALP and Runx2) by RT-PCR method. The undifferentiated cells were used as control in RT-PCR (scale bar = 40X).

BMSCs like other MSCs had the capacity to adhere to culture plastic flasks, and morphologically, cells appear as spindle-shaped cells resembling fibroblasts (Fig 2A–2C). Calculation of PDT showed that cell doubling time for BMSCs was 25.23 h (Fig 2D). This time is approximately the same as doubling time in other mesenchymal stem cells. Also, adipogenic and osteogenic differentiation of BMSCs was evident in Sudan III and Alizarin red staining, respectively. In brief, at the end of adipogenesis, Sudan III was used to stain the lipid droplets. Also, redness of the nodules indicated the presence of mineralized compartments as a result of the osteogenic treatment following Alizarin red staining (Fig 2E and 2F). The gene expression of PPAR-α and PPAR-γ as adipocyte-specific genes as well as ALP and OCN as osteocyte-specific genes was confirmed with RT-PCR, respectively.

### Immunophenotypic characterization of MSCs derived bone marrow

**Fig 3:**
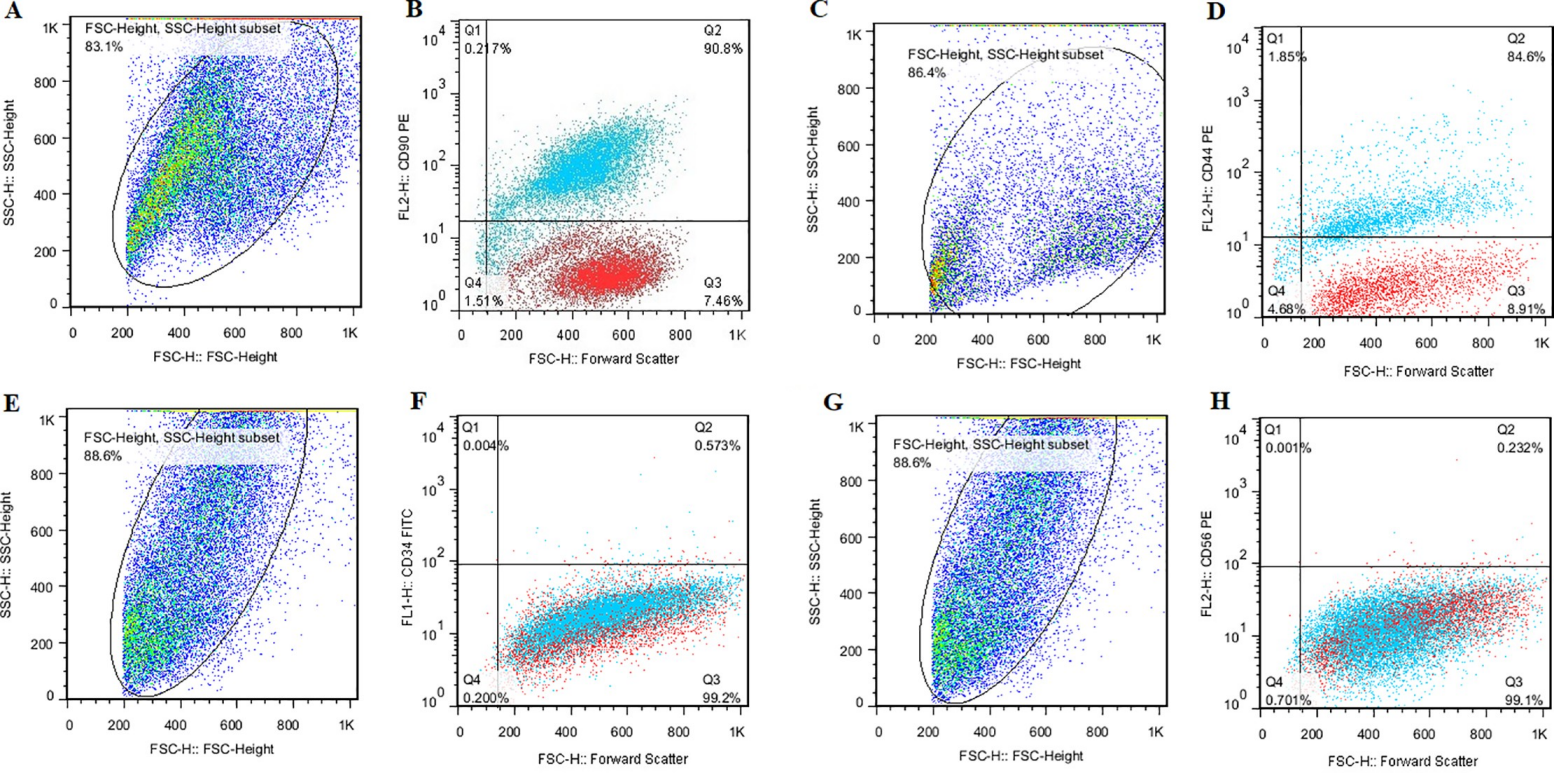
The expression of the cell surface markers of bone marrow derived-MSCs that analyzed by flow cytometry. Each cell surface marker was tested individually by separate cell population distributions and the isotopes controls were used as the negative control in this experiment; (A) a total population of cells for CD90 evaluation, (B) the bone marrow derived-MSCs were positive for CD90 (90.8%), (C) a total population of cells for CD44 evaluation, (D) the bone marrow derived-MSCs were positive for CD44 (84.6%), (E) a total population of cells for CD34 evaluation, (F) the bone marrow derived-MSCs were negative for CD34 (0.57%), (G) a total population of cells for CD56 evaluation, (H) the bone marrow derived-MSCs were negative for CD56 (0.23%). For CD34 and CD56, the isotype control was mouse IgG1 and for CD90 and CD44, the isotype control was mouse IgG2b. Also, isotype control is seen with red dots.

The cells (BMSCs) were analyzed for expression of a panel of cell surface markers as shown in Fig 3. The results revealed that BMSCs were positive for mesenchymal markers CD90 (90.8%) and CD44 (84.6%), but negative for hematopoietic markers CD34 (0.57%) and CD56 (0.23%).

### The cytokine secretion profile of K562 cell line co-cultured with bone marrow derived-MSCs

**Fig 4:**
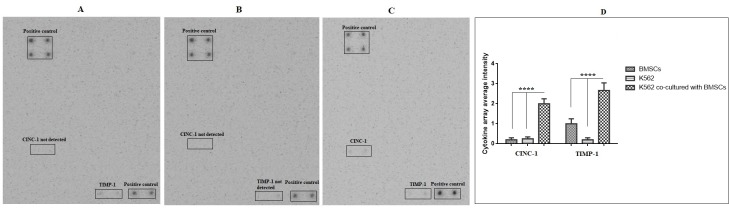
Antibody array analyses of cytokines present in bone marrow derived-MSCs. (A) Cytokines presence in culture medium of bone marrow derived-MSCs alone, (B) Cytokines presence in culture medium of K562 alone, (C) Cytokines presence in culture medium of bone marrow derived-MSCs co-cultured with K562 cells, (D) Quantification of cytokine array; (****P < 0.0001).

The cytokine antibody array membrane incubated for 2 hours with a fresh culture medium as suggested in the manufacturer’s protocol yielded a number of spots whose intensities were significantly stronger than the background level, indicating that culture medium of BMSCs (control group) contains soluble factors that could be cross-reactive to antibodies. However, when the array membrane incubated with a BMSCs culture medium was treated by K562 cell line (experimental group), the hybridization signals for most of these serum proteins were maximized to background. As common to the experimental and control groups, the arrays featured a single predominant hybridization signal for TIMP-1 (tissue inhibitor of metalloproteinases-1), and moderate elevated signals for CINC-1 (cytokine-induced neutrophil chemoattractant-1) (Fig 4A and 4B). To facilitate further analyses, all spots in the arrays were semi-quantified and their net intensity values were obtained by subtracting the background intensity. Among 34 cytokines implemented in the array, only two cytokines have the spot intensity above 100 while the intensity for a majority of other cytokines remained at the background level (Fig 4C). This observation indicates that only a small subset of cytokines may be abundantly secreted in co-culture medium of BMSCs and K562 cell line.

### Cell cycle kinetics regulation of K562

**Fig 5:**
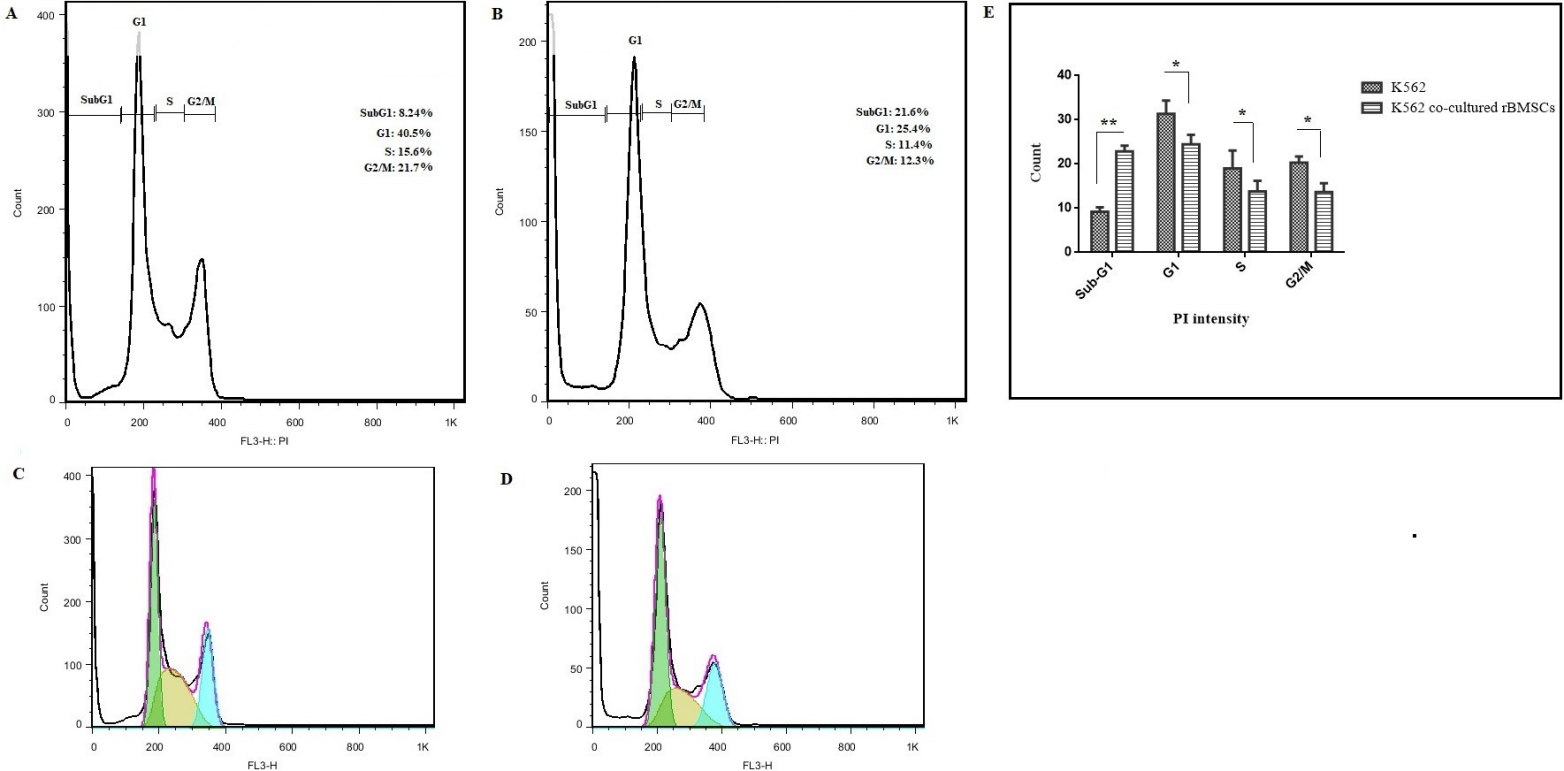
**(A-D) Representative image of cell cycle distribution.** A and C are cell cycle distribution of K562 cells alone (control group); B and D are cell cycle distribution of K562 cells co-cultured with bone marrow derived-MSCs (experimental group). **(E) Quantification analysis of cell cycle distribution;** (*P < 0.05 and **P < 0.01 compared with control group).

The cell cycle distribution of K562 cell line-being exposed to BMSCs revealed a significant increase in the percentage of G_0_/G_1_-phase (subG_1_) in comparison to the control group (10.22 ± 2.2% versus 24.19 ± 7.1%). Also, G1, S and G2/M phase fractions were decreased by 25.69 ± 8.80% versus 21.05 ± 2.1%, 31.02 ± 1.8 versus 16.22 ± 9.64 and 18.94 ± 3.01 versus 12.45 ± 7.86, respectively. Our data confirmed that BMSCs could change the pattern of K562 cell cycle to near-normal levels (Fig 5). In other words, by comparing the cell cycle distributions of the K562 cell line co-culture with BMSCs with those from the control culture, it was possible to estimate the cell cycle selectivity in the transition from the live to the apoptotic cell compartment.

### Investigation of apoptosis percentage by Annexin V/PI assay

**Fig 6:**
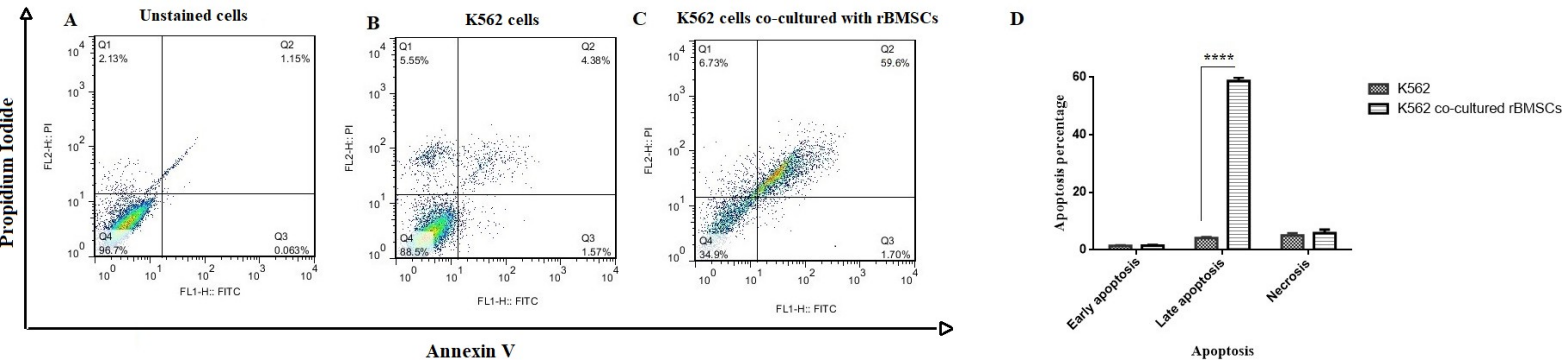
Flow cytometric analysis of bone marrow derived-MSCs co-cultured with K562 cells was performed with a combination of Annexin V-FITC, propidium iodide (PI). A shift from bottom-right quadrant panel (early apoptosis) to top-right quadrant panel (late apoptosis) and top-left quadrant panel (necrosis) was observed. (A) Unstained cells, (B) Control group and (C) Experimental group.

As we know, early apoptotic cells are Annexin V positive and PI negative (Annexin^+^, PI^-^) and late apoptotic or necrotic cells are positive to both Annexin V and PI (Annexin^+^, PI^+^). In order to assess the effect of BMSCs on apoptosis, K562 cell line was co-cultured with BMSCs for 7 days. Fig 6 shows the contour diagrams of Annexin V and PI stained K562 cells using flow cytometry after the 7^th^ day of co-culture with BMSCs. In other words, as illustrated in Fig 6, early apoptosis (Annexin^+^, PI^-^) was only 1.70% for K562 cell exposed with BMSCs. While about 59.6% of the cells were in late apoptotic stage (Annexin^+^, PI^+^), which was 13.6 times higher than that of the control group (4.38%) (Fig 6B–6D). The results suggest that in the presence of BMSCs, apoptosis in K562 cell would occur at a significant level.

### Bone marrow derived-MSCs reduced K562 cell viability via caspase3

**Fig 7:**
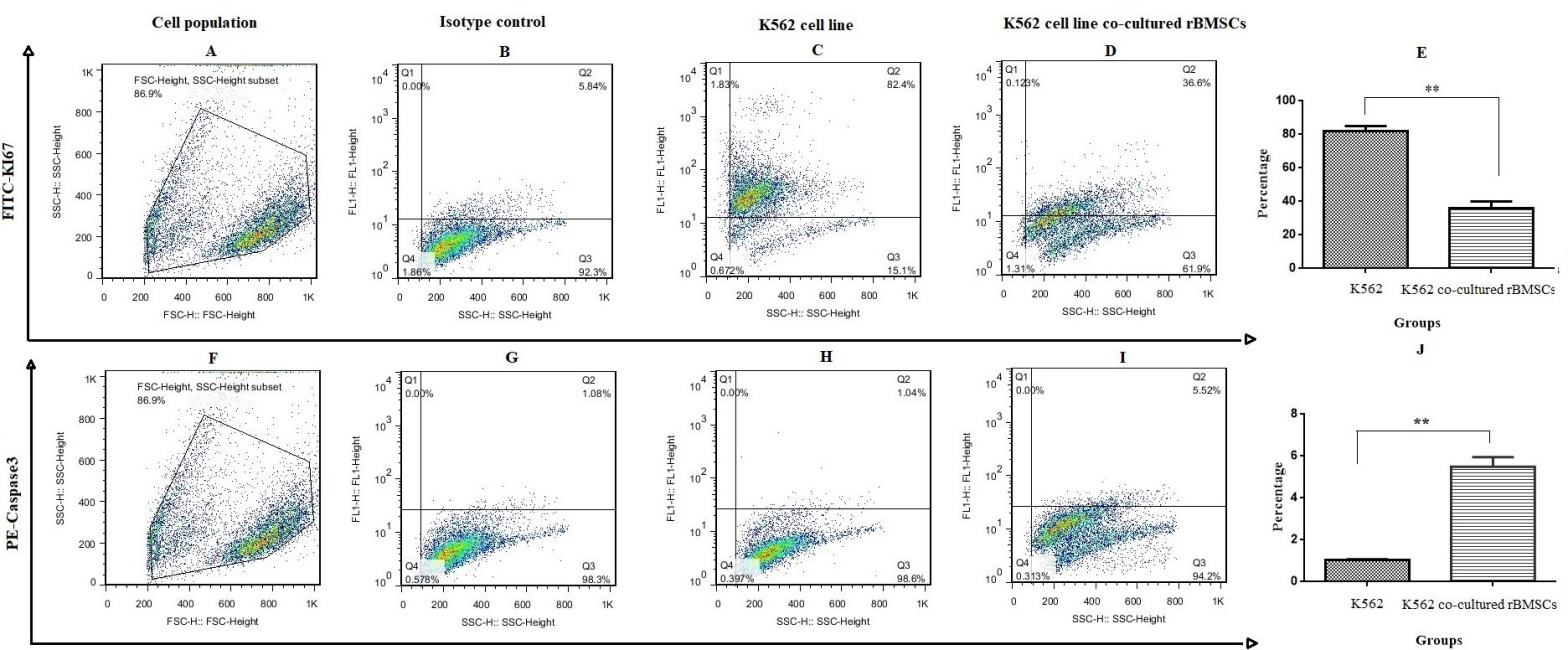
Proliferation and apoptosis of K562 cell line following co-culture with bone marrow derived-MSCs. Harvested cells were evaluated with Ki-67 (A-E) and apoptosis (F-J) by flow cytometry. In this figure, A and F are selected cell population, B and G are isotype control, C and H are K562 cells alone, finally D and I are K562 cells co-cultured with bone marrow derived-MSCs. Values are mean ± SD from independent experiments; (**p<0.01).

The predominant effect of bone marrow derived-MSCs on inhibition of cell proliferation was detected by Ki-67 expression (Fig 7A–7E). It was established that the downregulation of Ki-67 (proliferative surface marker) in co-cultured cells in comparison to the control group in which 7 days after co-culture, the percentage of Ki-67 in the control cells reached 45.8% was attributed to bone marrow derived-MSCs (Fig 7D and 7E) (**p<0.01). Also, apoptosis of K562 cells was evaluated following co-culture of these cells with MSCs by caspase-3 assessment. This part of the results showed that caspase-3 level was increased by about 5% (Fig 7I and 7J) (**p<0.01).

### Bone marrow derived-MSCs effect changed the gene and protein expression of Bax and BCL-2 in K562 cell line

**Fig 8:**
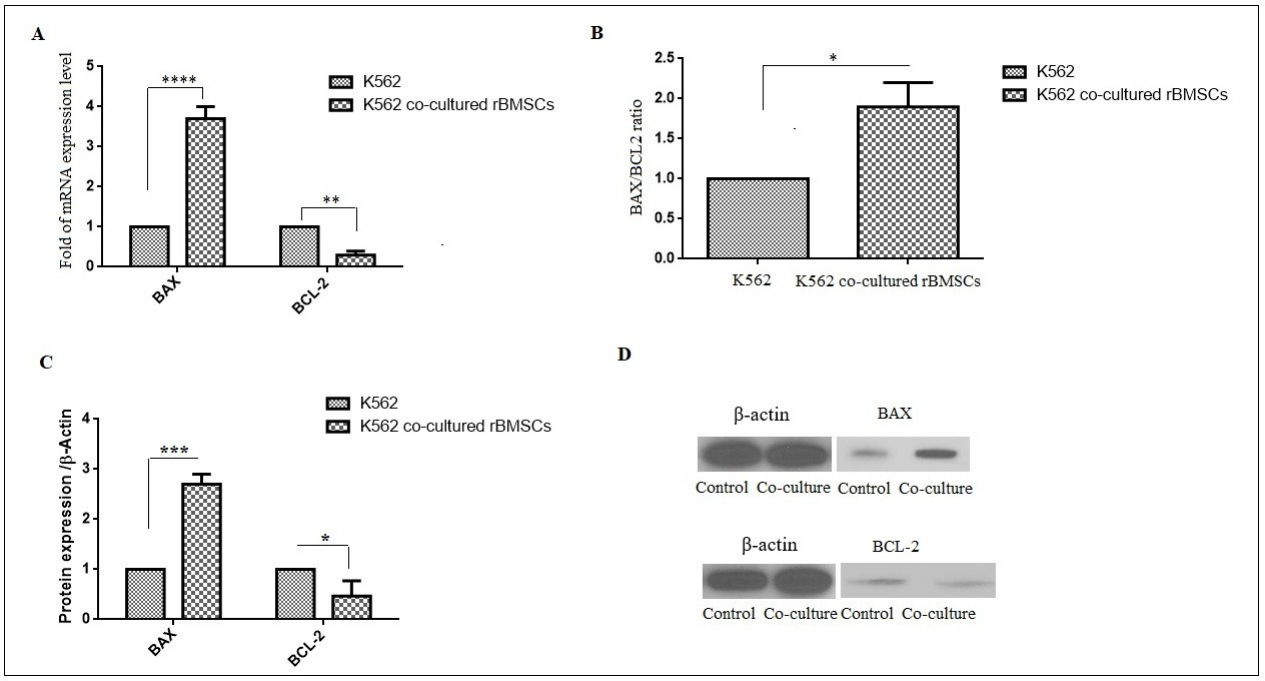
Effect of bone marrow derived-MSCs on apoptosis related gene and protein expression. (A) The mRNA and (C and D) the protein expression levels of BAX, BCL-2. (B) Bax/Bcl-2 ratio. The data presented as mean±SD of 3 independent experiments. *p< 0.05; ***p< 0.001 and ****p< 0.0001.

For evaluating the effect of cytokines secreted from BMSCs on pro- and anti- apoptotic agents of chronic leukemia cell line, the mRNA and protein expression was examined by real time-PCR and western blot, respectively. In this panel, BAX as a pro–apoptotic and BCL-2 as an anti-apoptotic elements were investigated. As shown in Fig 8A, the expression of BAX and BCL-2 mRNA levels was significantly increased and decreased by about 5.7 and 0.3 folds, respectively. In addition, the apoptotic BAX/BCL-2 ratio was increased by 1.9 folds in co-cultured K562 cell line (Fig 8B). In the following, the protein expression levels of BAX and BCL-2 were significantly changed. In other words, the level of BAX protein significantly increased when the level of BCL2 protein was significantly decreased in co-cultured K562 cell with BMSCs (Fig 8C and 8D).

## Discussion

Cancer is one of the major causes of morbidity and mortality throughout the world. Unlike the various therapeutic strategies such as radiotherapy, chemotherapy, and surgery, these approaches are often limited by the recurrence of metastasis, drug resistance, off-target effects or complications caused by these methods [27]. With this explanation, stem cell-based therapy as an alternative treatment has attracted researchers and clinicians [28]. Stem cells as unique population, are defined by their ability to: self-renew, differentiate into various cell types and form single cell-derived clonal cell populations [29]. Stem cells can be generally categorized as adult or somatic stem cells and embryonic stem cells (ESCs). Adult stem cells, which are generally multipotent cells and can differentiate into any cell type with a specific lineage, including endothelial progenitor cells (EPCs), hematopoietic stem cells (HSCs), neural stem cells (NSCs), MSCs, and others [30]. Among the various types of stem cells, MSCs are more considered in stem cell-based therapy. MSCs are a population of pluripotent cells that can proliferate and differentiate into mesenchymal lineage populations (bone, fat, cartilage etc.). MSCs-derived different tissues are used as novel therapies in regenerative medicine, grafting, scar remodeling and functional restoration of tissues [31]. Disease considered to be candidates for cell-replacement therapy including neurodegenerative disease, spinal cord injury, stroke, immunotherapy, inflammatory bowel disease, liver disease, diabetes, bone disease, chronic wounds, sepsis and respiratory diseases [32, 33]. In the last decade, MSCs as well as some classes of progenitor cells have been widely studied as one of the most suitable candidate seed cells for repairing and regenerating cardiomyocytes as well as restoring heart function [34]. Therapies engaging stem cells are showing increasing promise in the cancer treatment and anticancer drug screening applications. Stem cells can function as novel delivery platforms by homing to and targeting both primary and metastatic tumor foci [35]. Stem cells engineered to stably express various bioactive factors decrease tumor volumes in preclinical animal models [36]. There is abundant evidence that different types of MSCs could abolish tumor growth *in vitro* and *in vivo*. For example, Secchiero et al. (2010) indicated that BMSCs could inhibit tumor growth in immunodeficient mice bearing disseminated non-Hodgkin’s lymphoma xenografts [28]. Furthermore, in another study, it was shown that umbilical cord matrix stem cells completely attenuated rat mammary adenocarcinoma with no evidence of metastasis or recurrence [37]. Anti-tumour and anti-proliferative effects of adipose tissue derived-MSCs (ADSCs) were also reported. Cousin et al. (2009) reported that intra-tumoral injection of ADSCs in a model of pancreatic adenocarcinoma inhibited tumor growth [38]. Also, it was found that ADSCs inhibited the growth of human U251 glioma cells *in vitro* [39]. In the following, Yang et al. (2014) also found that the growth of lung cancer cell line A549, rectal cancer cell line HT29, and breast cancer cell line MCF-7 was inhibited by ADSCs [39]. Despite the inhibitory effects of MSCs on tumour cells, contradictory information has also been reported. It was demonstrated that MSCs derived from any kind of connective tissue and bone marrow provide a microenvironment for growth, survival, and differentiation of both normal and leukemic hematopoietic cells [40, 41]. Furthermore, it was reported that BMSCs population seems to be important in leukemogenesis and also contribute to chemoresistance through its release of specific soluble mediators [42, 43]. In another study, Sun et al. (2008) reported that BMSCs played an important role in proliferation and tumor angiogenesis of melanoma cells [44].

Because bone marrow microenvironment is necessary for repopulation of normal hematopoietic stem cells, studies related to BMSCs are on the rise. BMSCs do not express co-stimulatory molecules, such as CD40, CD40L, B7 and so on, or express only a low level of MHC-II antigens which are related to stimulation of immune system or immune recognition [45]. Therefore, BMSCs as well as adipose tissue derived-MSCs have attracted the interest of many researchers in the fields of tissue engineering, cell transplantation and cancer therapy [46]. Also, whether isolated cells are true MSCs is very necessary for the reliability of the experimental results. Studies have shown that the purity of MSCs obtained by density gradient separation with Ficoll-Hypaque in comparison with the cell attachment method can reach 95% at the first generation and exceed 98% at the second generation [47]. The high homogeneity of BMSCs in our study was consistent with previous reports. It is now well investigated that the main stem cell properties, including multi-lineage differentiation capacity, self-renewal and tissue engraftment, are principally influenced by the growth factors and cytokines of their own. A set of growth factors and cytokines may form a cytokine network which confers stability and flexibility to the cells, and rapid amplification of response against a special stimulus. It is well known that a variety of growth factors secreted from a neighboring MSC population largely contribute to cytokine network, but their molecular constituents and respective roles are yet to be well defined [48]. There are several growth factors and cytokines produced by MSCs which plays a key role in modulating cancer cells. In one study by Li et al. (2012), it was shown that MSCs can produce prostaglandin E2 (PGE2) after stimulation of IL-1a and IL-1b secreted by colon cancer cells. This stimulation leads to a secretion of IL-6 by MSCs which increase stemness properties of colon cancer cells [49]. In another study, it was reported that conditioned media derived-MSCs culture includes IL-6 able to induce expression of Oct4 and Sox2 as pluripotent markers in colorectal cancer stem cells (CSCs) [50]. In fact, two mentioned studies demonstrated that cytokines secreted from MSCs cause to strong expansion of CSCs and promotes also proliferation and invasion of cancer cell lines. These findings contradict with hypothesis of our study. Other cytokines secreted by MSCs can be IL4, IL8, IL10, IL17b, CXCL1, CXCL5, 6 and 7 and EGF. The pattern of secreted cytokines and chemokines by MSCs is strictly dependent on tumor cell types and niches [51].

Therefore, in this study, we aimed to determine the cytokine secretion profile of BMSCs co-cultured with K562 cell line using a cytokine antibody array that could analyze simultaneously protein expressions of up to 34 cytokines. The overall spectrum of this array contains MSC-secreted cytokines, such as GM-CSF, IL-6, IL-8, MCP-1 and PDGF. However, it is better to use the kind of array that is broad enough to contain most of the MSC-secreted cytokines like the one reported by Park et al. (2009) [48]. However, when the array membranes were incubated with secretion media of the BMSCs in three groups (control group, BMSCs alone, and experimental group, BMSCs co-cultured with K562 cell line), the expressed hybridization signals were only observed for two cytokines; TIMP-1 and CINC-1 and the majority of other cytokines were hybridized at zero or minimal level. Similar phenomenon was observed in the study of Park et al., (2009), where only IL-6, IL-8, MCP-1, TIMP-2, VEGF and OPG through 120 cytokines were identified in BMSCs [48]. These results indicated that the rest of the cytokines shown in Fig 4 are not expressed in BMSCs; this finding is new and valuable. In addition to the cytokine array, cell cycle distribution assay and Annexin V/PI staining were used. As reported in previous studies, fluorescent probe Annexin V/PI is commonly used for the quantification of apoptosis because it binds to phosphatidylserine exposed on the surface of apoptotic cells. On the other hand, the identification and management of the mechanism of cell damage/death induced by cytokines and growth factors secreted by MSCs is particularly important in the assessment of the biological reaction to cell therapy. In line with Annexin V/PI results, it was well-determined that the rate of late apoptotic cells was profoundly increased in which 4.38 late-apoptotic cells in the control group eventually reached 59.6% after co-cultured with BMSCs. In addition, the level of necrotic cell clearly shifted from 5.55 to 6.73% 7-day post co-cultured.

Different studies have been done on the effect of various MSCs-derived tissues on cancer cell cycle distribution. Comparison of cell cycle progression of K562 cultured in experimental and control groups showed that there was an accumulation predominantly in G_0_/G_1_ phase (Fig 5), slowing entering into S phase. In other words, BMSCs are attributed to a robust increase in the number of cells at G_0_/G_1_ phase that implied cell arrest at G_0_/G_1_ phase. This result agrees with earlier reports by different authors [16, 17]. Also, in line with another investigation, it can also be concluded that BAX, as well as caspase-3 cascade, has a critical role in the arresting of cell cycle distribution. Along with all the results obtained, we hypothesized that the change in the cell cycle distribution, as well as, induction of apoptosis by BMSCs was governed by cytokines. Liu and Hwang (2005) and Park et al. (2009) had also used the cytokine antibody array to analyze cytokine secretion by umbilical cord blood derived-MSC, they found that IL-6, IL-8 and TIMP-1 were the most abundant proteins expressed partly, which is consistent with our data showing that the TIMP-1 and CINC-1 were expressed in culture media of BMSCs [48]. The two prominent cytokines detected in co-culture most probably are derived from BMSCs as BMSCs culture alone indicates a high secretion of these cytokines (2.02-fold and 5.11-fold increase for TIMP-1 and CINC-1, respectively). In this study, we did not detect cytokines such as IL-6, IL-8, VEGF, TIMP-2, MCP-1 and OPG, as reported by another study [48]. But interestingly, we are the first to report that the expression of CINC-1 cytokine was undetectable in cultured media of BMSCs, but detected in co-culture media of K562 cell and BMSCs.

## Conclusion

Looking at present evidence, there is still no clear information about the effect of MSCs on cancer cells, due to contradicting effects that could be favorable or unfavorable for cancer cell growth. Unfortunately, this process is complicated by cellular interactions between MSCs and cancer cells that include membrane fusion, metabolites or growth factors that shape the relationship of MSCs with tumor cells. In this content, there is no doubt that caution should be taken in the field of cell based-therapy when MSCs is used in patients with cancer history. In the other words, if cancer cells survive following surgery, they will probably induce resident MSCs to promote tumor angiogenesis, thus causing to tumor growth. With all these interpretations, the results of this study showed that BMSCs led to a significant induction on apoptosis and cell cycle arrest of CML-cell line. Cell-cycle arrest was exerted by halting the progression of K562 cells in G_0_/G_1_ phase. Hypothesizing that cytokines and growth factors might be highly involved in the anti-tumor effect mediated by BMSCs, we analyzed the cytokine secretion profile. Significant production of TIMP-1 and CINC-1 by BMSCs was found, and was quantified by normalizing the array spots. The physiological role of TIMP-1 and CINC-1 remains unclear. The significant expression of TIMP-1 and CINC-1 cytokines in co-culture media of K562 cell and BMSCs suggests that these cytokines could be involved in the inhibition of the tumor cell proliferation via BAX and caspase-3 cascade. The identity of another molecule involved in the anti-proliferative effect of BMSCs requires further investigation.
